# Willingness-to-pay in physical activity: how much older adults value the community-wide initiatives programs?

**DOI:** 10.3389/fpubh.2023.1282877

**Published:** 2023-10-31

**Authors:** Sittichat Somta, Marc Völker, Dyah Anantalia Widyastari, Sirinapa Mysook, Narakorn Wongsingha, Danusorn Potharin, Piyawat Katewongsa

**Affiliations:** ^1^Thailand Physical Activity Knowledge Development Centre (TPAK), Salaya, Nakhon Pathom, Thailand; ^2^Institute for Population and Social Research, Mahidol University, Salaya, Nakhon Pathom, Thailand; ^3^Sisaket Provincial Public Health Office, Mueang, Sisaket, Thailand

**Keywords:** community-wide initiatives, older adults, physical activity, well-being, willingness-to-pay

## Abstract

**Background:**

Previous studies have shown insufficient physical activity (PA) as a significant global health concern and a major risk factor for non-communicable diseases (NCDs). Community-wide initiatives in physical activity (CWIPA) is considered as a best-buy for Community-wide initiatives in physical activity (CWIPA) is considered as a best-buy for NCDs prevention. However, assessment regarding resource allocation and cost-effectiveness of existing programs is lacking. This study investigated local residents’ willingness-to-pay (WTP) for community PA programs in Southern Thailand.

**Methods:**

The contingent valuation method (CVM) using the payment card approach was employed to elicit the WTP of 472 residents aged 45 years and over in selected provinces in which community PA programs had been implemented. Respondents were asked to indicate their WTP for the continuous offering of free CWIPA by choosing how much they were willing to pay from eight bid-value options, payable through their monthly electricity bill.

**Results:**

The mean WTP of Thai older adults was found to be 72 baht/month ($2/month) or 868 baht/year ($25/year). This indicated the maximum amount an older person was willing to pay for any community-based PA program. More than half the sample (54.2%) chose zero as their answer, while there was a fairly large variation in other levels of WTP. The WTP was lower among older respondents and those who resided in rural areas but was higher among those with a history of participation in an organized PA program.

**Conclusion:**

The level of WTP can be interpreted as an indicator of community satisfaction with CWIPA. That finding can be used as evidence for the government and policy makers in allocating resources and designing future CWIPA. A variety of organized PA programs should be offered to all community members to ensure inclusivity and also to provide equal access for senior citizens.

## 1. Introduction

The change of the demographic structure, accompanied by a rise in the proportion of older adults and the progression toward an aging society is attributed to a decline in mortality rates and an upward trend in life expectancy (1). Unfortunately, these trends are also accompanied by increases in the prevalence of non-communicable diseases (NCDs) and disability in old age, affecting the cost of acute and long-term care (2). More and more countries are confronting the challenge of escalating medical expenses among their older populations, particularly regarding the cost of healthcare for individuals with NCDs. By 2030, it is projected that the worldwide prevalence of newly diagnosed NCDs and adverse mental health conditions will reach approximately 500 million individuals, leading to an estimated healthcare expenditure exceeding $30 billion per year (3).

Previous studies have shown insufficient physical activity (PA) as a significant global health concern and a major risk factor for NCDs (3–7). Accordingly, more countries are now actively promoting PA among the older population, particularly since it can ameliorate symptoms and comorbidities associated with over 26 NCDs (4, 8). Promoting PA for older adults also contributes to enhancing body function, reducing impairment, supporting independent living, and improving the overall quality of life (9–11). Despite the documented advantages of PA, the prevalence of sufficient PA among older adults worldwide has remained at a sub-optimal level (57%) in recent years (12, 13).

PA participation is threatened by the inequality of access and opportunity (14, 15). In many settings, older persons experience barriers in accessing PA amenities, and have less opportunity to engage in regular PA compared to younger adults (16). That said, the home community can serve as a focal point for PA promotion, aiming to ensure equal access and participation. Promoting PA within the community setting therefore, can effectively enhance and sustain the PA levels of older adults (17–20). Thailand, as one of the countries that has transitioned into an aging society, places significant emphasis on promoting PA to safeguard the health of its older population. To facilitate this, the Community Health Security Fund (CHSF) established by the National Health Security Office (NHSO) plays a pivotal role in advancing PA promotion at the community level, thereby enhancing and expanding participation in PA across all age groups. Despite the ongoing efforts in many countries (including Thailand) to promote PA, there remains a dearth of comprehensive assessments regarding the return on investment in PA promotion initiatives.

The lack of economic evaluation of the cost–benefit of investment in PA means that there is insufficient information for effective planning and decision-making. To address this gap, the “Willingness to Pay” (WTP) indicator has become a popular method in the economic field to estimate the value individuals assign to goods or services in non-market contexts (21). WTP represents the maximum amount individuals are willing to pay for a particular investment in a specific scenario (22). Understanding the level of WTP is highly valuable for policymakers and healthcare providers when making decisions on resource allocation within budgetary constraints (21, 23, 24). WTP also serves as a crucial tool for assessing the monetary value of health outcomes and estimating the cost-effectiveness of interventions (25).

Throughout the past 5 years, the CHSF has supported a community-wide initiative program in PA (CWIPA). It is one of an array of investment policies to promote PA among senior citizens community, with the initial purpose to raise awareness and organize PA within the home community. However, there has been a lack of assessment regarding resource allocation and cost-effectiveness in these programs. Therefore, the aim of this study is to investigate WTP for CWIPA, and identify the factors that influence WTP in CWIPA. The results of the study should provide valuable information and guidelines for the local and central government in allocating limited resources toward promoting community-based PA, particularly for the older adults.

## 2. Methods

### 2.1. Participants

The analysis draws on a contingent valuation method (CVM) study of public preferences for PA programs in three provinces of southern Thailand. The selection of the three provinces was based on whether any PA projects had been implemented continuously for at least 3 years in the province, and whether clear details of these activities were available on the website of the CHSF of the NHSO. We identified the communities where PA promotion programs were available, and defined the community members in those communities as the population of the study. From the specific areas of which the sample of survey participants was drawn, it was further narrowed down to four, then we randomly-selected sub-districts, two urban and two rural. We drew our sample randomly from the population list of community members who participated and who did not participate in the CWIPA. A total of 472 residents aged 45 years and over were involved in the study.

### 2.2. Data collection

A structured questionnaire was used to collect data on respondents’ (i) valuation of PA programs, (ii) participation in PA programs, (iii) perception of health, social and environmental outcomes of participating in PA programs, and (iv) basic demographic and socioeconomic characteristics. Personal interviews were conducted by field staff who were well-trained to avoid interviewer bias.

The CVM was applied to elicit a respondent’s valuation of PA programs in their community. In a CVM study, respondents are directly asked about their WTP for the provision of nonmarket goods or services, such as community-based PA programs. CVM has been widely used in studies aimed at valuing the nonmarket social benefits of public sector investment in various fields of research, such as environmental conservation by Han et al. (26), health economics by Liu et al. (27), tourism by Hakim et al. (28), and PA by Herens et al. (29). A variety of question formats can be used to elicit a respondent’s WTP in CVM. In this research, the payment-card format was employed which asks respondents to choose the amount that best reflects their WTP from a defined set of options. Compared to other approaches, the payment-card format is relatively easy to implement, and is robust with small sample sizes (30).

In this study, respondents were asked to indicate their WTP for the continuous offering of free public aerobics, *“ba-salope”* (Lao line dance) and other types of dance classes, and the continuous maintenance of public outdoor gyms in their home community. Respondents could choose how much they were willing to pay from eight bid-value options (in Baht): 0, 25, 50, 75, 100, 125, 150, or above 175, payable through their monthly electricity bill. They were instructed to select the next-lowest option in case their true WTP was between any of these bid-value s (e.g., respondents with a true WTP of 30 baht should choose 25 baht). The bid-value options were derived from a pre-test survey that was conducted on April 2, 2023, in order to mitigate possible anchoring bias which is an inherent risk in studies that rely on the payment-card approach. Acceptance of using a surcharge to the monthly electricity bill as the payment vehicle was also verified through the pre-test. To reduce the risk of hypothetical bias, a “cheap talk” script^1^ was added prior to the valuation question that reminded respondents of the hypothetical nature of the above scenario and explained that respondents often tend to overstate their WTP under this condition. The valuation question was followed up by a set of questions aimed at identifying uncertain and protest responses, as well as cases of scenario rejection. Other sections of the questionnaire captured respondent participation in PA programs, perception of health, social and environmental outcomes of participating in PA programs, and demographic and socioeconomic characteristics.

### 2.3. Data analysis

Multivariate regression analysis was used to identify correlates of the sample’s WTP, assess the validity of the study, and derive an estimate of mean WTP. The choice of the type of regression model used to estimate the WTP function depends on the scale of the dependent variable. When conducting a CVM study using the payment-card approach, a respondent’s unobservable true WTP may lie between the chosen bid-value and the next highest bid level. One way of constructing the dependent variable from these intervals is to calculate the midpoint as an approximation of the true WTP, and that method was the approach taken in this research (30). It is important to note that the upper bound of the highest interval is subject to assumptions, as the highest bid-value on the payment card was unbounded (above 175 baht). In this study, the two highest intervals were combined, and the upper bound of the combined interval was specified as the highest bid-value plus one unit, which is a conservative approach to handle the unbounded highest bid-value in the analysis (31). A Tobit model was used to estimate the WTP function. Given that the midpoints of the bid-value intervals constitute a continuous dependent variable, an ordinary least squares model was initially considered. However, due to the observed censoring of WTP at the lowest bid-value, the use of a linear model would have resulted in biased and inconsistent parameter estimates (32).

The Tobit model was developed by Tobin (33) and its general form can be written as


$$Y_{i}=\beta_{0}+X_{i}\beta_{j}+\mu_{i};\mu_{i}\sim N\left(0,\sigma \right);\forall i=1,2,3,\ldots ,n$$

$$W_{i}=Y_{i}\;if\;Y_{i}\geq L$$

$$W_{i}=L\;if\;Y_{i}<L$$

where $W_{i}$ is the WTP of respondent i according to the chosen bid-value level, $Y_{i}$ is an unobserved latent variable to which $W_{i}$ is hypothetically related, $\beta_{0}$ is the regression intercept, $X_{ji}$ is a set of independent variables (j = 1,…), $\beta_{j}$ is a vector of corresponding regression parameters, $\mu_{i}$ is the stochastic error, i are the survey respondents, N(0,σ) denotes the standard normal distribution with zero mean and constant variance σ, and L is the lower limit of the dependent variable (here: the lowest possible bid-value). In this study, the set of independent variables $X_{i}$ captures basic demographic and socioeconomic respondent characteristics as well as respondents’ participation in PA programs.

Subsequently, the mean of respondents’ WTP was calculated using the following equations.


$$E\left(Y\right)=\Phi \left(\frac{\beta \bar{X}}{\sigma }\right)\left(\beta \bar{X}+\sigma \lambda \right)$$

$$\lambda =\frac{\phi \left(\frac{\beta \bar{X}}{\sigma }\right)}{\Phi \left(\frac{\beta \bar{X}}{\sigma }\right)}$$

where E(Y) is the predicted mean value of WTP, Φ(.) is the cumulative distribution function of the standard normal distribution, ϕ(.) is the probability density function of the standard normal distribution, β is the coefficient vector of the independent variables, $\bar{X}$ are the mean values of the vector of the independent variables, and σ is the standard error of the error term.

In the final step of the analysis, the aggregated total WTP for community PA programs was calculated by multiplying the mean WTP estimate obtained from the Tobit model by the valid number of residents aged 45 years or older in the study area. Given the uncertain true WTP of those survey respondents who were found to provide protest answers, or who rejected the hypothetical contingent valuation scenario, or were unsure about their answer, a sensitivity analysis including two scenarios was conducted. In the first scenario, the population size used in the calculation of the total WTP was the number of all residents aged 45 years or older, while in the second, more conservative, scenario this population size was multiplied by the share of survey respondents who provided valid answers to the WTP question (i.e., no protest answers, scenario rejection, or ambivalence).

### 2.4. Ethical approval

The data collection tool and procedures complied with local and national regulations. Participants were informed of the purpose of the study, and that their participation was completely voluntary. They were informed that they could choose to respond or not to respond to the questions for any reason. They indicated their approval to be involved in the study by signing their consent before data collection commenced. The protocol for this study received ethical approval from the Institute for Population and Social Research Mahidol University Thailand, with registration number COA. No. 2023/02–021.

## 3. Results

Table 1 provides an overview of demographic and socioeconomic characteristics of the sample. A total of 472 respondents were interviewed, comprising 11% male and 89% female, with 43% age 45–59 years and 57% aged 60 years or older. Most respondents (62%) were married, 21% widowed, 12% single, 4% divorced/separated, and 2% did not reveal their marital status. In terms of educational attainment, 38% had completed only primary education, 32% secondary education, and 30% tertiary education. One out of three respondents operated a private business, 31% were unemployed, with the remaining respondents having other occupations or providing no answer. The income distribution shows that the vast majority of respondents had an average monthly income in the range of below 3,500 baht to 30,000 baht, with a minor share having either a higher income or no income at all. Due to the sensitive nature of income information, 15% of respondents chose not to reveal their average monthly income. Over half the sample had been diagnosed with a disability/NCDs, out of which 41% had difficulty in performing activities of daily living (ADL).

**Table 1:** Demographic and socioeconomic characteristics of survey respondents (*n* = 472).

Variable	Total sample (%)
Sex	
Male	52 (11%)
Female	420 (89%)
Age group (years)	
45–59	205 (43%)
60 or older	267 (57%)
Marital status	
Single	59 (12%)
Married	291 (62%)
Widowed	97 (21%)
Divorced/separated	18 (4%)
No answer	7 (2%)
Education	
Primary	178 (38%)
Secondary	152 (32%)
Higher	140 (30%)
No answer	2 (0 + %)
Occupation	
Private enterprise	155 (33%)
Formal employee	76 (16%)
Informal employee	46 (10%)
Agriculture	39 (8%)
Unemployed	146 (31%)
Health volunteer	8 (2%)
No answer	2 (0 + %)
Income	
No income	20 (4%)
Below 3,500 baht	80 (17%)
3,500–10,000 baht	151 (32%)
10,001–15,000 baht	63 (13%)
15,001–30,000 baht	48 (10%)
30,001–50,000 baht	23 (5%)
50,001–100,000 baht	13 (3%)
100,001–300,000 baht	2 (0 + %)
No answer	72 (15%)
Disability/NCDs	
Yes	248 (53%)
No	224 (47%)
Difficulties with ADLs	
Yes	102 (41%)
No	146 (59%)
Not applicable	224

NCDs, non-communicable diseases; ADLs, Activities of Daily Living.

In terms of their WTP for CWIPA, more than half the sample (54.2%) chose zero as their answer, while there was a fairly large variation in other levels of WTP (Table 2). Around one in seven (14.4%) were willing to pay 100 baht, followed by 9.3% who were willing to pay 175 baht or more, 10% who were willing to pay 50 baht, 9.1% who were willing to pay 25 baht, and a minor share of other responses. As mentioned earlier, the selected WTP bid-values only approximate a respondent’s true WTP, which may be equal to or higher than the selected bid but lower than the next (not selected) higher bid. Out of those respondents who stated a WTP of zero, a debriefing question revealed that two-thirds made that choice because their true WTP lay between 1 and 24 baht, while 7.8% stated that they could not afford any additional monthly payment (Table 3).

**Table 2:** Respondents’ WTP for CWIPA programs (*n* = 472).

WTP (in baht)	Frequency	%
0 (0–24)	256	54.2
25 (25–49)	43	9.1
50 (50–74)	47	10.0
75 (75–99)	6	1.3
100 (100–124)	68	14.4
150 (150–174)	8	1.7
125 (125–149)	0	0.0
175 or more	44	9.3
Total	472	100.0

**Table 3:** Debriefing questions (*n* = 472).

Variable	Frequency	%
How sure are you that you would be willing to pay this amount in a real situation? (if WTP > 0)		
Very unsure	0	0.0
Unsure	12	2.5
Sure	106	22.0
Very sure	100	21.0
Not applicable (WTP = 0)	254	54.0
When you decided on your WTP, did you believe that your community authorities will be successful in ensuring the continuous offering of community PA programs?		
Yes	335	71.0
No	137	29.0
When you decided on your WTP, did you agree with the idea that you will pay through your monthly electricity bill?		
Yes	234	50.0
No	238	50.0
Do you think the city government would use the results of this study to set new tariffs and implement the new plan?		
Yes	300	64.0
No	172	36.0
What is the main reason why you are not willing to pay anything?		
I cannot afford the additional monthly payment	20	7.8
I am not sure about the sustainability of the plan	6	2.3
I do not like the additional charges to be added to my electricity bill	21	8.2
I do not like our community authorities or other government authorities managing these community PA programs.	2	0.8
I do not see the need for funding for the continuous offering of community PA programs	18	7.0
It is the government’s responsibility, not mine, to pay for the community PA programs	20	7.8
The informant can pay support from 1 to 24 baht	187	73.0

*n* = 472, except for last question which was only asked of respondents who chose the zero WTP bid level (*n* = 256). WTP, willingness to pay; PA, physical activity.

A number of respondents were uncertain about their WTP, gave a protest answer, or rejected the hypothetical valuation scenario. A minority of responses were coded zero if the respondent gave a protest response, i.e., said they were not willing to pay anything because of their uncertainty regarding the sustainability of the plan, disliked being charged through their electricity bill, disliked community or government authorities, disagreed with the need for funding to support PA, or felt that it is the government’s responsibility to pay for CWIPA programs (Table 3). Moreover, only a very small percentage of respondents were unsure that they would be willing to pay their selected amount in the real situation. However, a small yet noticeable share of the respondents did not believe that their community authorities would be successful in ensuring the continuous implementation of CWIPA programs when stating their WTP. Furthermore, half the sample did not agree with the payment vehicle. Around a third of the respondents did not think that their city government would use the results of this study to set new tariffs and implement a new plan for CWIPA programs (Table 3).

The estimation results of the Tobit models used to determine the factors correlated with WTP for CWIPA programs are shown in Table 4. Model 1 was estimated based on data from all respondents (without missing values in any of the included variables). In Model 2, cases of uncertainty, protest answers, and scenario rejection were excluded. The pseudo R2 values indicate a better explanatory power of the reduced sample Model 2 compared to the full sample Model 1. At the same time, the estimation results, in terms of the identified statistically-significant coefficients and their sign, are largely consistent and stable across the two models, with the exception of difficulties with ADL due to disability/NCDs which was only significant at the 10% level in Model 1 and lost its significance altogether in Model 2.

**Table 4:** Tobit regression results on WTP for CWIPA programs.

Variable	Model 1				Model 2			
	Coefficient	*P* > |t|	95% CI		Coefficient	*P* > |t|	95% CI	
			Lower	Upper			Lower	Upper
Age	−1.337*	0.06	−2.73	0.05	−2.121**	0.02	−3.91	−0.33
Gender	8.728	0.68	−32.61	50.07	−3.249	0.91	−58.68	52.18
Education	3.322	0.68	−12.45	19.09	−10.442	0.28	−29.63	8.75
Monthly income	0.001**	0.02	0.00	0.00	0.002**	0.01	0.00	0.00
Marital status								
Married	6.799	0.70	−27.66	41.26	−18.341	0.37	−58.74	22.05
Divorced/separated	31.009	0.14	−10.72	72.74	8.793	0.73	−41.49	59.08
Widowed	11.651	0.70	−47.82	71.12	17.796	0.62	−52.49	88.08
Difficulties with daily activities	−28.564*	0.05	−57.53	0.40	−4.961	0.78	−40.38	30.46
Participation in PA programs	58.271***	0.00	32.57	83.97	60.357***	0.00	28.20	92.51
Occupation								
Formal employee	−4.158	0.83	−41.31	32.99	3.527	0.89	−47.57	54.62
Informal employee	−29.400	0.16	−70.53	11.73	−28.762	0.36	−90.93	33.41
Agriculture	−23.058	0.34	−70.62	24.50	−14.843	0.57	−66.99	37.30
Unemployed	−7.731	0.60	−36.67	21.20	6.426	0.70	−26.66	39.51
Health volunteer	27.492	0.52	−56.09	111.08	−2.138	0.95	−76.29	72.01
Rural	36.201***	0.01	−61.79	−10.68	−46.845***	0.00	−78.02	−15.67
Intercept	70.721	0.30	−62.99	204.44	217.151***	0.01	59.72	374.58
Estimated variance	9255.796				5490.060			
Pseudo R2	0.0251				0.0340			
Number of observations	395				156			

Model 1 = Full sample; model 2 = Reduced sample without cases of uncertainty, protest answers, and scenario rejection. ***p < 0.01; **p < 0.05; *p < 0.1.

The factors that were found to be significant predictors of WTP for CWIPA in both models were age, income, previous participation in a CWIPA program, and living in a rural area. Older respondents were found to have a lower WTP. Respondents with a higher monthly income had a higher WTP, which is in line with economic theory (34). Previous participation in a CWIPA program was found to have a positive effect on WTP. Residency in a rural area was negatively associated with WTP. Overall, these results indicate that WTP varies in logical ways with respondent characteristics, which supports the face validity of the WTP estimates obtained in this study.

Table 5 presents the mean and total WTP estimates derived from the Tobit model estimation results. The mean WTP for the continuous offering of CWIPA programs from the reduced-sample Tobit model (Model 2) was estimated to be 72 baht/month (US$2/month) or 868 baht/year ($25/year). Multiplying the mean WTP estimate with the number of residents aged 45 years or older resulted in an estimated total WTP per district that ranged from around 21 to 103 million baht/year, or 620,000 to $3 million/year. Taking a more conservative estimation approach and multiplying the mean WTP estimate only by the valid number of residents aged 45 years or older (as described in the methods section), the estimated total WTP per district lies between 8 and 39 million baht or 233,000 and $1.1 million/year.

**Table 5:** Mean and total WTP for CWIPA programs.

	WTP (in baht)	WTP (in US$)
Monthly mean per resident	72	2
Annual mean per resident	868	25
Total annual per district		
*Scenario 1 (full population 45+)*		
District A	21,410,837	620,058
District B	26,750,527	774,695
District C	102,690,084	2,973,905
*Scenario 2 (adjusted population 45+)*		
District A	8,029,064	232,522
District B	10,031,448	290,511
District C	38,508,781	1,115,214

WTP, willingness to pay; PA, physical activity.

## 4. Discussion

The community-wide initiative in PA (CWIPA) has been known to be an effective strategy to reduce inequality in PA (17). While PA participation is largely differentiated by individual capacity and capability to access PA, studies have shown that successful CWIPA programs were able to improve population-level PA in general (35–37). Hence, CWIPA is also limited to certain communities which can afford the program, or where the government provides some assistance. In Thailand, the NHSO has been supporting CWIPA since 2018. With a total funding of approximately 45 million baht ($1.2 million) per year, selected communities received approximately 30,000–50,000 baht ($800–1,500) per year. Nevertheless, a declining trend in the proportion allocated for CWIPA was observed (8.4% in 2018, 8.7% in 2019, 7.2% in 2020, 6.0% in 2021, and 5.8% in 2022) (38).

Focusing on the inability of NHSO to finance CWIPA in the future as a hypothetical scenario, this study found 54.2% of all respondents selected a WTP of zero (response coded 0–24 baht). Out of these 54.2, 73% were willing to pay between 1 and 24 baht. Higher WTP levels were selected by 45.8% of all respondents. A low WTP in this study does not necessarily mean that CWIPA is regarded to have low value by the community members. Instead, it could be interpreted as rejection of the scenario, disagreement with the payment vehicle, disagreement with the need for funding because they believe it is the government’s responsibility to pay for CWIPA, or simply the low affordability of the community. These factors were considered in the subsequent estimation of mean WTP. A low WTP could also be seen as the reflection of the status quo during the past 5 years, where many communities have utilized the NHSO funding for their PA programs, with little/no contribution out of their pocket. The findings of this study also indicate that income had a positive association with WTP in both Tobit models, where increasing income will also increase the WTP. The significance of income in predicting WTP in PA programs has also been reported by many scholars, e.g., Gottschalk et al. (22), Herens et al. (29), and Romé et al. (39) which indicate that the valuation of PA programs is differentiated by respondent socioeconomic status (SES).

Age was negatively associated with WTP. This study found that older respondents had a lower WTP or PA. This could be attributed to physical disability/NCDs or strained finances that limit PA participation. A permanent disability may inhibit a person’s motivation to participate in a PA program, and that undoubtedly increases with age (40). That finding is also supported by the results from multivariate analysis where individuals who experience difficulty in performing ADL had lower WTP. Deterioration of the skeletal structure and muscle mass when entering old age leads most seniors to have a lower level of PA (41, 42). In addition, in another study, low PA participation was associated with lower WTP among older adults (43). Buying power of older people also generally decreases as a person leaves the work force and enters retirement (41).

Respondents who reside in rural areas have lower WTP compared to their counterparts in urban areas. While cost of living is usually significantly lower in rural Thailand compared to the city, it is also true that disposable income is often less for farmers than urbanites, and that could lead to generally lower WTP for goods and services in the countryside. Thai rural households are also worse off for multiple SES indicators, particularly for education (44). One study found that, other things being equal, rural Thais were more likely to engage in work-related (agriculture) PA than recreational PA (45). It should be noted that CWIPA programs in Thailand mostly offer recreational PA.

The history of participating in a PA program was the strongest determinant of WTP in this sample of Thais. Individuals who participated in CWIPA in the previous year were more likely to have higher WTP for organized PA. This result is consistent with the finding of previous studies that WTP is positively correlated with prior participation in leisure-time sport (43). Engaging in such activities generates positive net benefits which increase WTP after taking part in the program (29, 43). Higher WTP among older people who participated in CWIPA indicates a high awareness of community members of the importance of PA for their health, and that the existing programs have met their expectations and preferences. Previous studies found that motivation to participate in CWIPA was mostly driven from satisfaction toward the program, including the variety, time, cost, and social interaction with their peers (29, 43). Those factors may also increase WTP.

The mean WTP of Thai older persons in this sample was 72 baht/month (US$2/month) or 868 baht/year (US$25/year). This means that one older person in Thailand is willing to pay 72 baht/month or 868 baht/year for CWIPA. The annual WTP for CWIPA of Thai older person is somehow lower compared to other settings, i.e., in Netherland (US$120) (29), Finland (US$79) (46), Sweden (US$1,080) (47) or other developed nations. This finding implied that the community members’ perception on the value of the PA program in their community and the valuation was not only affected by their income, but also the differentiated by sociocultural and demographic characteristics of the individuals, such as age, area of residence, and whether or not they had participated in a CWIPA program in the previous year. The sociocultural and contextual setting where the study has taken place may help to explain the differentiation on WTP across countries with different culture and levels of development. While it is true that Europeans may be willing to pay more, CWIPA in Thailand are currently partially funded by the government. Thai people’s perception toward the importance of PA and their current level of PA (or participation) may also be different from their European counterparts. Thus, transfers of WTP estimates between countries (benefit transfer) should be made with caution and by considering the context of each study setting. Apart from the actual behavioral intention (WTP for the intervention, i.e., CWIPA), it should be noted that WTP could also be affected by respondent dissatisfaction toward the CVM (48). This study found that half the sample did not agree with having the payment for PA added to their monthly electricity bill, and about a third expressed ambivalence toward setting new tariffs and the implementation of new plan for CWIPA. However, some respondents agreed to the lower bid-values because they believed that such a scenario might actually happen, and would they eventually have to pay for participation in a CWIPA program. Hence, this study considered both types of response by employing two scenarios in the Tobit models. The consistency of results in both models indicated face validity of the WTP estimates of this study.

The present findings serve as the first evidence of WTP in CWIPA, and this information can be used to assist the government in making decisions, particularly in allocating resources for PA in the community. The extent of the variance in WTP indicates different valuations of CWIPA, with a high bid-value indicating satisfaction with the existing program, and also their affordability to purchase participation in the program. The results of the study can be used as an indirect evaluation of community-based interventions in PA, as seen from the community’s perceived benefit of the program. The difference in WTP by age, income, area of residence and PA participation also suggests which group of the population value CWIPA more and, thus, future interventions should be tailored to address the needs of the groups with lower WTP.

Several limitations of the study should be acknowledged. First, this study only covered the WTP of CWIPA based on how much the community values the investment. Future studies need to compare the benefits of PA with the costs in order to calculate the return of CWIPA on the investment. This study was not able to establish the actual cost because there is no reliable data on how much the government has invested in CWIPA *per capita* per year. There were also a variety of programs in each study site, and that made it difficult to estimate the actual value of a single CWIPA program, which could lead to inaccuracy in a cost–benefit comparison. Even though CWIPA, as a national policy, has been implemented nationwide, the number of areas with sustained PA activities is limited (i.e., only in the southern region). Therefore, national estimates for cost–benefit of CWIPA are difficult to establish at present. Second, this study focused exclusively on a limited number of provinces in the southern region of Thailand. Similar surveys need to be conducted in other parts of the country in order to obtain a comprehensive picture of how people value CWIPA, given that preferences and financial resources are likely differ between geographic regions. Third, most of CWIPA in the study involved programs (i.e., traditional dance, aerobic) where females were the majority of participants. Future studies should involve more varieties in the CWIPA that involved males. Finally, this study only investigated the WTP of adults aged 45 years or older. Future studies need to include younger cohorts who might also participate in and gain benefit from community-based PA programs.

## 5. Conclusion

The mean WTP of Thai older adults is 72 baht/month ($2/month) or 868 baht/year ($25/year). This may indicate the maximum amount an older person can afford to pay for any community-based PA program; 7.8% were not willing to pay for CWIPA at all. The WTP was lower among the older age segment of the sample and those who resided in rural area, but was higher among those with a history of participation in an organized PA program. The level of WTP is an indicator of community satisfaction with CWIPA. That finding can be used as evidence for the government and policy makers in allocating resources and designing future CWIPA. A variety of organized PA programs should be offered to all community members to ensure inclusivity and also to provide equal access for senior citizens.

## Data availability statement

The datasets presented in this study can be found in online repositories. The names of the repository/repositories and accession number (s) can be found at: https://tpak.or.th/th/article/712.

## Ethics statement

The studies involving humans were approved by Institute for Population and Social Research Mahidol University Thailand, with registration number COA. No. 2023/02–021. The studies were conducted in accordance with the local legislation and institutional requirements. Written informed consent was obtained from the individual (s) for the publication of any potentially identifiable images or data included in this article.

## Author contributions

SS: Conceptualization, Data curation, Formal analysis, Investigation, Writing – original draft. MV: Conceptualization, Data curation, Formal analysis, Methodology, Writing – review & editing. DW: Conceptualization, Investigation, Supervision, Writing – original draft, Writing – review & editing. SM: Resources, Writing – original draft. NW: Resources, Writing – original draft. DP: Resources, Writing – original draft. PK: Conceptualization, Funding acquisition, Supervision, Writing – review & editing.

## Footnotes

^1^Use of a “cheap talk script” entails reading a script that explicitly highlights the hypothetical bias problem before a study participant makes any decision.
